# Escape Room vs. Traditional Assessment in Physiotherapy Students’ Anxiety, Stress and Gaming Experience: A Comparative Study

**DOI:** 10.3390/ijerph182312778

**Published:** 2021-12-03

**Authors:** Guadalupe Molina-Torres, Irene Sandoval-Hernández, Carmen Ropero-Padilla, Miguel Rodriguez-Arrastia, Jesús Martínez-Cal, Manuel Gonzalez-Sanchez

**Affiliations:** 1 Department of Nursing, Physiotherapy and Medicine, Faculty of Health Sciences, University of Almería, 04120 Almería, Spain; guada.lupe@ual.es; 2 Department of Physical Therapy, Faculty of Health Sciences, Campus of Melilla, University of Granada, 52005 Melilla, Spain; isandoval@ugr.es; 3 Pre-Department of Nursing, Faculty of Health Sciences, Jaume I University, 12071 Castello de la Plana, Spain; arrastia@uji.es; 4 Department of Physiotherapy, Faculty of Health Sciences, University of Málaga, 29071 Málaga, Spain; mgsa23@uma.es; 5 Biomedical Research Institute of Malaga (IBIMA), 29010 Málaga, Spain

**Keywords:** physiotherapy, escape room, evaluation, students, higher education

## Abstract

Escape Rooms can serve multiple academic and educational purposes and can be used as part of the evaluation of a learning program. The aim of this study was to analyze the levels of anxiety and stress perceived in the evaluation using the Escape Room compared to the traditional evaluation, as well as to analyze the gaming experience. Methods: A comparative study was carried out in students of the Degree in Physiotherapy, with a total of 56 participants who underwent 2 evaluation processes. The variables analyzed were the State—Trait Anxiety Inventory, the Perceived Stress Questionnaire, and the Gaming Experience Scale. A comparative analysis was performed between the groups using the Mann Whitney U test and Student’s T test. Results: The levels of state-anxiety and trait-anxiety were higher in the traditional assessment group. Although no differences were found in the qualification obtained by the students, statistically significant differences were found between the two evaluation systems in terms of the overload factor, the energy factor, and the fear-anxiety factor of the perceived stress questionnaire. Conclusions: The Escape Room can be considered as an alternative to the traditional evaluation in Physiotherapy Degree students due to its lower levels of anxiety and perceived stress.

## 1. Background

The involvement of students in teaching-learning activities requires the commitment of both the student and the teacher, depending on some factors, such as the goals and expectations of both, the teacher’s support, and educational and motivational practices [1]. There are several resources to encourage student participation and active learning in higher education, such as problem-based learning [2], flipped learning [3] and gamification [4], which place the needs of the students at the center of the teaching-learning process. Specifically, gamification can stimulate student interest and motivation, increase interaction among students, and promote collaborative learning [5,6]. Thus, gamification, in general, and Escape Rooms, in particular, can help students to consolidate knowledge, acquire new skills and develop specific attitudes in a controlled virtual reality that favors learning [7]. Escape Rooms are increasingly popular in Higher Education [8] as a complement to traditional teaching methods [9], allowing students to learn by doing, that is, learning through lived experiences and reflecting on day-to-day problems, both academic and clinical [10]. The original Escape Rooms were conceived as a game, in which a team of players cooperatively discover clues, solve puzzles and complete tasks in one or more rooms to progress and achieve a specific goal [9]; with the same approach, it is used as a learning tool [11]. The activity is largely centered on a story or case that explains the general purpose of the activity, in which participants must collaborate to solve specific topic-related puzzles and riddles in a limited period of time in order to meet predetermined objectives [12]. Usually, there are several challenges that must be solved for the team to progress through the game until it is completed and thus escape from a room or situation [13]. The literature shows that Escape Rooms in educational settings are novel ways to encourage collaboration, problem solving and communication [14,15,16], having a positive impact on the learning process and being a novel method for delivering interactive teaching [17]. In most cases, a time limitation is introduced as an element of stress, excitement and competition [18].

The implementation of educational games helps to increase participation and knowledge among students [19], as well as to evaluate the theoretical-practical contents, interprofessional communication, teamwork, and practical skills [20]. Escape Rooms can serve multiple academic and educational purposes and can be used as part of an assessment of a learning program [9]. The Escape Room as an evaluation method could be especially important due to its multiple benefits, mainly the reduction in stress, the improvement of motivation, a better identification of strengths and weaknesses, and the improvement of professional practice and decision making [20,21,22]. Several studies have included the use of the Escape Room as an evaluation method in nursing students [5,23]; however, there are no studies in which the Escape Room tool is used in the teaching-learning process or as an evaluation system in Physiotherapy students. Therefore, the aim of this study was to analyze the implementation of the Escape Room as an evaluation method in students of the Degree in Physiotherapy compared to the traditional evaluation, in order to observe the levels of anxiety and perceived stress, as well as to analyze the gaming experience among these students.

## 2. Methods

### 2.1. Study Design

**Figure 1:**
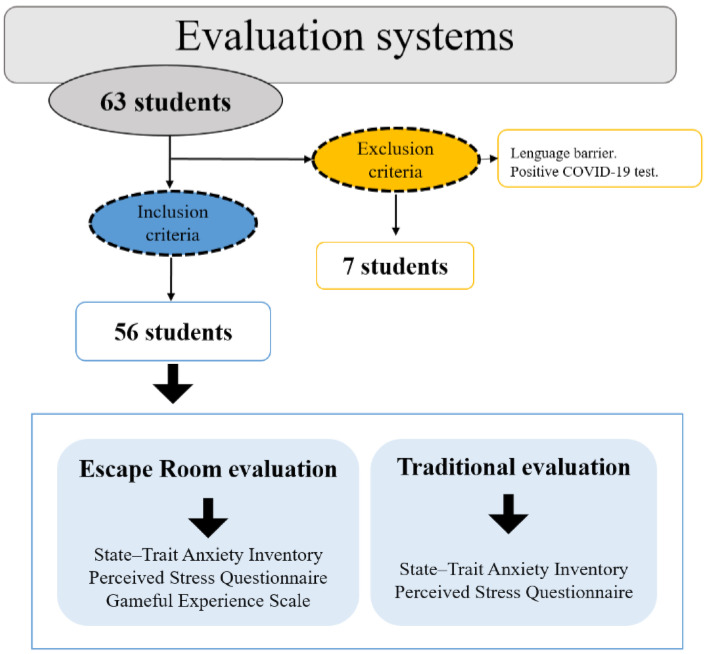
Flow diagram of participants.

A comparative study was carried out in students of the Degree in Physiotherapy, with a total of 63 participants of which only 56 who underwent two evaluation processes on different days and 7 students were excluded. In terms of earlier preparation time costs, the Escape Room required about 7 h to design and fully set up, and the traditional evaluation consumed approximately 3 h. Firstly, the traditional evaluation was carried out and, the next day, the evaluation was carried out by the Escape Room. All of the students had the same time to carry out both types of evaluation. The inclusion criteria were the following: (a) being over 18 years old, and (b) being enrolled in the subject of General Procedures in Physiotherapy I. On the other hand, the exclusion criteria were the following: (a) students with an insufficient level of Spanish to be able to perform both tests normally and (b) a positive COVID-19 test that made it impossible to participate in the Escape Room. All of the students participated in the two evaluation processes (Figure 1).

### 2.2. Ethical Considerations

The students were informed about the objective of the study, and of the confidentiality and anonymous treatment of the data. Before data collection, the study was approved by the Ethics Committee of the Spanish public university with protocol number EFM 132/2021 and the data were used in accordance with Organic Law 3/2018, of December 5, on the Protection of Personal Data and guarantee of digital rights. The ethical principles set out in the Declaration of Helsinki were also followed. Subsequently, the participants signed the informed consent.

### 2.3. Setting and Participants

This study was carried out in a Spanish public university. The participants were students of the Degree in Physiotherapy enrolled in “General Procedures in Physiotherapy I”, which is a compulsory subject of 6 European Credit Transfer and Accumulation System (ECTS) credits, and it is taught in the second semester of the first year. This subject consists of theoretical and practical classes where the students are organized in groups of 8–10. The content of this subject introduces students to the generalities of physical agents, introduction to massage therapy, thermotherapy, cryotherapy, hydrotherapy, and movement as a therapeutic resource.

### 2.4. Instruments

After recording the sociodemographic characteristics of the students, the following assessment instruments were used:

*State–Trait Anxiety Inventory (STAI)*: This is a questionnaire that includes 40 items designed to evaluate 2 independent concepts of anxiety: on the one hand, anxiety as a state (transitory emotional condition) and anxiety as a trait (relatively stable anxious propensity). Each of the subscales (state anxiety/trait anxiety) is composed of a total of 20 items in a 4-point Likert response system according to intensity (0 = almost never/not at all; 1 = something/sometimes; 2 = quite a lot/often; 3 = a lot/almost always). The total score in each of the subscales ranges from 0 to 60 points. In samples of the Spanish population, levels of internal consistency have been found to oscillate, both for the total score and for each of the subscales, between 0.84 and 0.93 [24].

*Perceived Stress Questionnaire (PSQ):* it measures stress in psychosomatic clinical research [25] and consists of 30 items scored with a Likert-type scale from 1 (almost never) to 4 (almost always). It also has 5 dimensions: tension -factor, social conflict factor, overload factor, energy factor, and fear-anxiety factor. Internal consistency was measured with the alpha coefficient, which was 0.9 [26]. PSQ index was obtained according to the indications of Levenstein et al. [27], i.e., PSQ = (raw score_30/90).

*Gaming Experience Scale (GAMEX):* it measures the gaming experience among Physiotherapy students during the Escape Room [28,29] and it contains 27 items that are scored using a Likert-type scale with a range from 1 (never) to 5 (always). At the same time, the 27 items are divided into 6 dimensions, which include enjoyment, absorption, creative thinking, activation, absence of negative effects, and dominance. The total Cronbach’s α value was 0.855 [29].

### 2.5. Traditional Evaluation

The traditional evaluation consisted of an examination, in pairs, of the practical procedures of the subject, scoring from 0 to 10 in each of the four cases according to the correct position of the patient, the position of the Physiotherapist students with respect to the patient and the execution of the technique (safety, speed, depth, etc.) (see Appendix A, Table A1).

### 2.6. Escape Room Evaluation

**Figure 2:**
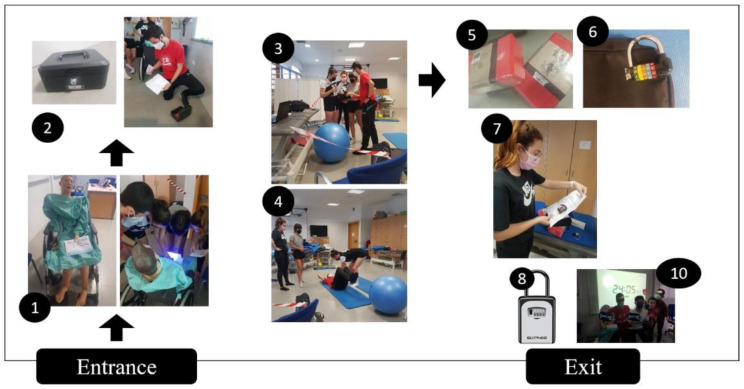
Escape room with physiotherapy students.

In the evaluation through the Escape Room, each student had to solve a clinical case individually and the help of all the members of the group was required to open the locks and boxes that led from one case to another in order to escape from the room. Each group consisted of 4 people, and they had 30 min to escape. The four clinical cases were solved with the contents taught in the practical classes of the subject. The items that made up the individual evaluation of each student included the position of the patient, the position of the Physiotherapist students, the indications and corrections that the Physiotherapist students gave the patient and the procedures proposed based on the contents of the subject, with a score range of 0 to 10. In the first case, for example, students were required to perform the procedure to release the diaphragm in order to collect the materials required to unlock the first padlock and gain access to the following case (Figure 2).

### 2.7. Procedure

All of the students first took the traditional assessment and then took the assessment by the Escape Room as part of the compulsory assessment of the subject with official grades within the official schedule. The students recruited for the study were those enrolled in the subject of general procedures in physiotherapy I. Furthermore, a single teacher evaluated all of the students, both in traditional evaluation and through the Escape Room. At the end of the traditional evaluation, the students completed the anxiety and perceived stress questionnaires. Moreover, at the end of the Escape Room evaluation, they completed the anxiety, perceived stress, and gaming experience questionnaires. The questionnaires were completed in approximately 10–15 min and the anonymity of their responses was guaranteed.

### 2.8. Statistical Analysis

Firstly, a descriptive analysis of the results was carried out, calculating the measures of central tendency and dispersion for the quantitative variables, while, for the categorical variables, the frequency and the percentage were analyzed. To make the comparison between the groups, and according to their distribution, the Mann Whitney U test and Student’s *T*-Test were used for the non-parametric and parametric variables, respectively. A value of *p* < 0.05 was considered significant. SPSS version 25 statistical software was used for the data analysis.

## 3. Results

### 3.1. Sociodemographic Characteristics of the Participants

The sample consisted of 63 students of the Degree in Physiotherapy, of which 56 students met the inclusion criteria and were able to take part in both evaluation processes. Of the total sample included in the study, 26 were women (46.4%), and 30 were men (53.6%), with a mean age of the total sample of 20.02 ± 4.16 years, with the mean age of women being 19.73 ± 2.18 years and that of men 20.27 ± 5.38 years.

### 3.2. Analysis of the Acquisition of Practical Skills

Regarding the final grade for the acquisition of practical skills through traditional assessment, the mean was 8.66 ± 1.48, and, on the other hand, the final grade through the Escape Room was 8.47 ± 1.69, showing no statistically significant differences between the 2 groups.

### 3.3. State–Trait Anxiety Inventory (STAI) and Perceived Stress Questionnaire (PSQ)

The results obtained in relation to anxiety and perceived stress in each of the evaluation systems are detailed in Table 1. The levels of state-anxiety were higher in the traditional evaluation group, exhibiting statistical significant. Statistically significant differences were also found between the two evaluation systems in terms of the overload factor, the energy factor, and the fear-anxiety factor of the perceived stress questionnaire, with the levels of the overload factor being higher in the traditional evaluation system with respect to the evaluation by the Escape Room, as well as the levels of the fear-anxiety factor. However, energy factor levels were higher in the Escape Room assessment group.

### 3.4. Game Experience Scale (GAMEX)

The results obtained in the gaming experience scale are detailed in Table 2, where the scores obtained in each of the dimensions of the GAMEX in relation to the evaluation through the Escape Room are specified on the total number of participants.

## 4. Discussion

The aim of this study was to analyze the levels of anxiety and stress perceived in the evaluation using the Escape Room compared to the traditional evaluation, as well as to analyze the gaming experience. Once the results were analyzed, it was observed that the level of practical skill demonstrated in each of the tests did not present differences between the two groups. However, statistically significant differences were observed in state-anxiety with respect to the two types of evaluation, perceiving that anxiety levels are lower during the evaluation process developed through the Escape Room. At the same time, statistically significant differences were also found in perceived stress in terms of overload factor, energy factor and fear-anxiety factor between the two evaluation methods.

The evaluation system using the Escape Room shows lower levels in terms of state-anxiety levels, although these results cannot be compared with any study where two different evaluation systems are used. A study in which anxiety levels were measured before and after performing the Escape Room showed that anxiety levels decreased in the test carried out in the simulation laboratory [30]. One possible explanation is that students were working as a team, and despite its preparation, higher costs, and investment of time, the Escape Room could foster a more relaxed atmosphere than a traditional evaluation [20]. At the same time, the results obtained in relation to perceived stress cannot be compared with any other study in which the Escape Room is either proposed as an evaluation system or compared with a traditional evaluation system. However, a previous study suggests that changes in assessment regimes should always be evaluated to determine the impact on student learning outcomes and well-being [31].

On the other hand, from the results obtained in relation to the analysis of the gaming experience on the evaluation system through the Escape Room, the Physiotherapy students obtained the highest scores in enjoyment, absorption, creative thinking, activation, and dominance. In addition, the students reported very few negative effects of the gaming experience, which is in line with the results of other studies where activation and the absence of negative effects stand out [32,33], although these studies are focused on learning through the Escape Room and not on evaluation, as in the present study. On the other hand, the results of the present study can be contrasted with another study where the Escape Room is used as an evaluation method that offers high levels of enjoyment, absorption, creative thinking, activation and dominance [6,23]. In addition, it should be noted that no differences were found between the two evaluation systems in terms of the acquisition of practical skills, with similar scores being observed in the evaluation using the Escape Room. These results are not in line with a previous study carried out with nursing students, where significant differences were observed in the level of practical skills between the two evaluation systems in favor of the Escape Room [23].

### Strengths, Limitations and Future Lines of Research

It should be noted that there are no previous studies carried out with students of the Degree in Physiotherapy that compare different evaluation systems, including the Escape Room. The results of this study must be viewed in the context of several limitations. Firstly, the students who participated in this study were from a single Spanish university, and from a single year. Secondly, the degree of satisfaction and usefulness for the faculty members involved in the evaluation using the Escape Room was not measured, which would have allowed us to obtain an even deeper understanding of their level of satisfaction with this type of evaluation. That being said, these findings warrant further discussion, such as exploring physiotherapy educators’ perceptions, in order to gain a better understanding of the implementation of these types of evaluation approaches. Further research, moreover, should be carried out to measure the impact of this type of evaluation and thus allow planning and creating Escape Rooms with the aim of evaluating the students of the Degree in Physiotherapy.

## 5. Conclusions

The main conclusion that can be drawn from the present study is that, through the Escape Room as an evaluation strategy in physiotherapy students, the results obtained in the evaluation of practical skills are similar to those achieved with the traditional evaluation. However, the Escape Room reduces the levels of state-anxiety within the dimensions of perceived stress, thus reducing the levels of overload factor and fear-anxiety factor. In addition, the levels of gaming experience showed high scores in terms of enjoyment, absorption, creative thinking, activation, and dominance dimensions, thus it could be used as an alternative to the traditional method in the assessment of knowledge and acquired skills.

## Figures and Tables

**Table 1:** Comparison between traditional and the Escape Room assessment.

Variables		Group	M ± SD	*p* Value
State anxiety		Traditional evaluation	**50.93 ± 9.92**	***0.001***
		Escape Room evaluation	**43.43 ± 10.64**	
Trait anxiety		Traditional evaluation	37.18 ± 10.19	*0.697*
		Escape Room evaluation	36.38 ± 11.58	
PSQ		Traditional evaluation	47.12 ± 10.70	*0.548*
		Escape Room evaluation	45.83 ± 11.88	
PSQ	Tension factor	Traditional evaluation	48.41 ± 14.96	*0.457*
		Escape Room evaluation	46.16 ± 16.89	
	Social conflict factor	Traditional evaluation	33.84 ± 17.62	*0.960*
		Escape Room evaluation	34.01 ± 18.39	
	Overload factor	Traditional evaluation	**54.31 ± 22.36**	***0.025***
		Escape Room evaluation	**49.702 ± 20.16**	
	Energy factor	Traditional evaluation	**50.23 ± 22.77**	***0.022***
		Escape Room evaluation	**55.23 ± 19.88**	
	Fear-anxiety factor	Traditional evaluation	**59.52 ± 27.12**	***0.018***
		Escape Room evaluation	**50.29 ± 27.79**	

**M**: mean; **SD**: standard deviation. ***Bold and italics marks***: indicate statistical significance.

**Table 2:** GAMEX dimension: mean and standard deviation of total of participants.

Dimension	Total of Participants
	M ± SD (Range)
Enjoyment	26.43 ± 4.01 (6–30)
Absorption	23.09 ± 5.12 (6–30)
Creative thinking	15.96 ± 3.36 (4–20)
Activation	14.63 ± 2.72 (4–20)
Absence of negative effects	5.48 ± 2.85 (3–15)
Dominance	14.12 ± 2.66 (4–20)

**M**: Mean; **SD**: Standard Deviation. **Range** = lowest score-highest score achievable.

## Data Availability

The data that support the findings of this study are available on request from the corresponding author. The data are not publicly available due to privacy or ethical restrictions.

## Appendix A

**Table A1:** Evaluation rubric used for case 1 in traditional evaluation.

Case 1: Diaphragm Realease Produce				
Items	0.5	1	1.5	2
Position of the patient				
Position of the student				
Instructions and recommendations to the patient				
Proposed training for the patient				
Ongoing evaluation				
